# Assessment of clinical application of preimplantation genetic screening on cryopreserved human blastocysts

**DOI:** 10.1186/s12958-016-0155-z

**Published:** 2016-04-08

**Authors:** Min Liu, Yu Su, Wei-Hua Wang

**Affiliations:** Center for Reproductive Medicine, Changsha Hospital for Maternal and Child Health Care, No.416, Chengnan East Road, Changsha City, Hunan China; Houston Fertility Laboratory, 2500 Fondren Rd., Suite 350, Houston, TX 77063 USA

**Keywords:** Aneuploidy, Blastocyst, Cryopreservation, Implantation

## Abstract

**Background:**

Recent advancement in both human embryology and preimplantation genetic screening has created a completely new situation for human assisted reproduction. Embryos, typically at blastocyst stage, are biopsied and screened by DNA microarray or next-generation sequencing before cryopreservation, and then euploid embryos are warmed for transfer. Increased embryo implantation rates have been reported after transfer of euploid embryos screened for all chromosomes. However, some patients may have frozen their embryos without doing embryo biopsy and screening, thus embryo biopsy and screening may be required after cryopreservation and warming. Such procedures have not been performed routinely in clinics and the efficiency is still unknown. Therefore, in this study, we investigated embryo implantation after blastocysts were cryopreserved/warmed, and then biopsied and screened by DNA microarray for all chromosomes.

**Results:**

Two hundred and thirty four cryopreserved blastocysts from 35 women were warmed, and 224 (95.7 %) survived and were biopsied for aneuploidy screening. After analysis, 221 samples (98.7 %) had diagnostic results and 3 (1.3 %) samples did not have results due to DNA quality and quantity. Out of the samples with diagnostic results, 59.3 % were normal euploid and 40.7 % had abnormal chromosomes including aneuploidy, partial chromosome deletion and/or duplication. Most (65.6 %) samples had single chromosome anomalies, and 34.4 % of the samples had multiple chromosome anomalies. Chromosomal errors were observed in most chromosomes but chromosomes 21 and 22 had the most frequent chromosome anomalies. Transfer of 61 normal euploid blastocysts in 34 patients resulted in a 52.9 % clinical pregnancy rate and a 42.6 % implantation rate, and 41.2 % of the patients delivered normal babies or had ongoing pregnancy.

**Conclusions:**

Frozen blastocysts can be warmed and biopsied for aneuploidy screening. These results may suggest that cryopreserved blastocysts can be warmed, biopsied and screened the day before embryo transfer, and such procedures may benefit patients who had previous implantation failures, or patients who did not have embryo screening before cryopreservation due to lack of embryo screening technology or other reasons.

## Background

There are many reasons for patients to cryopreserve their embryos after in vitro fertilization (IVF), such as high risk of ovarian stimulation, endometrial development unsynchronized with the embryos, elevated progesterone and surplus embryos after transfer. Recently, due to application of preimplantation genetic screening (PGS) for aneuploidy by using array comparative genomic hybridization (aCGH) and next-generation sequencing (NGS) in human IVF, most embryos, especially blastocysts, are cryopreserved after biopsy for PGS. It has been found that frozen embryos had similar implantation rates as compared with fresh ones [1, 2] and such high implantation rates are contributed by the new cryopreservation technology: vitrification [3, 4], indicating that cryopreservation of human blastocysts by vitrification has caused almost no trauma to the embryos and their implantation potential is not affected by freezing/warming procedures.

Embryo implantation can be further increased after PGS by transferring normal euploid embryos [5, 6]. It is well known that women of advanced maternal age have a high risk of producing aneuploid embryos, resulting in implantation failure, a higher risk of miscarriage or birth defects [7–9]. It has been found that PGS especially benefits women with previous IVF failure(s), repeated miscarriages, and those of advanced maternal ages [10–12]. Blastocyst biopsy for PGS is thought be to the most efficient approach as multiple cells are biopsied from trophectoderm (TE) cells, which provides more accurate results as compared to cleavage stage embryo biopsy or polar body biopsy before and after egg fertilization [13–15].

Practically, most patients request biopsy and PGS before they start IVF cycles, thus embryos (blastocysts) are biopsied before cryopreservation. This is the most common procedure for PGS. However, some patients may request the biopsy and PGS after embryo cryopreservation. These patients include 1) young patients who thought that PGS was not necessary for them during a fresh IVF cycle but whose embryo implantation failed after fresh embryo transfer(s); 2) no PGS procedure was provided in the previous IVF cycles, so patients’ embryos were cryopreserved without biopsy and PGS; 3) patients’ IVF cycles were performed a few years ago (3 years ago or up to 30 years ago) and PGS by aCGH or NGS was not available at that time. For these reasons, some patients may request biopsy and PGS on their cryopreserved embryos before frozen embryo transfer (FET) allowing the patients to have normal embryos for transfer. Recently, we found that increased numbers of patients requested PGS for their frozen embryos. Although embryo biopsy has been applied to fresh blastocysts for a few years, it has not been evaluated in frozen blastocysts. No data is available about TE biopsy for PGS after blastocysts are cryopreserved and warmed. Therefore, in the present study, we evaluated the efficiency of biopsy and PGS of frozen/warmed human blastocysts by examining clinical pregnancy and embryo implantation rates.

## Methods

### Ethics

Patients undergoing IVF, and PGS signed written consents for all kinds of laboratory and clinical procedures. The data was retrospectively collected from the medical records and the study was approved by Institutional Review Board (NEIRB 14-504).

### Vitrification and warming

Blastocysts were vitrified by using Irvine vitrification kit described as a previous method [16, 17]. Briefly, all blastocysts were equilibrated in a warmed equilibration solution on a warming stage (37 °C) for 2 min, then transferred into a warmed vitrification solution and then loaded onto a vitrification straw between 40–60 s before vitrification in liquid nitrogen. The blastocysts were stored in the liquid nitrogen for at least one month.

Blastocysts were warmed in the morning one day before scheduled FET. Vitrified blastocysts were taken out of liquid nitrogen, and the straw tip with blastocyst(s) was immersed into 1.0 M warmed (37 °C) sucrose solution for 1 min, then blastocysts were transferred to 0.5 M sucrose solution at room temperature for 3 min and finally to a basic solution (no sucrose) for 10 min with a solution change after 5 min at room temperature. After warming, blastocysts were cultured in Global medium (IVFonline.com) supplemented with 10 % serum protein substitute at 37 °C in a humidified atmosphere of 6.5 % CO_2_, 5 % O_2_ and balanced nitrogen before biopsy. Blastocyst quality was assessed by using standard assessments developed by the Society of Assisted Reproductive Technology [18].

### Blastocyst biopsy

**Fig. 1:**
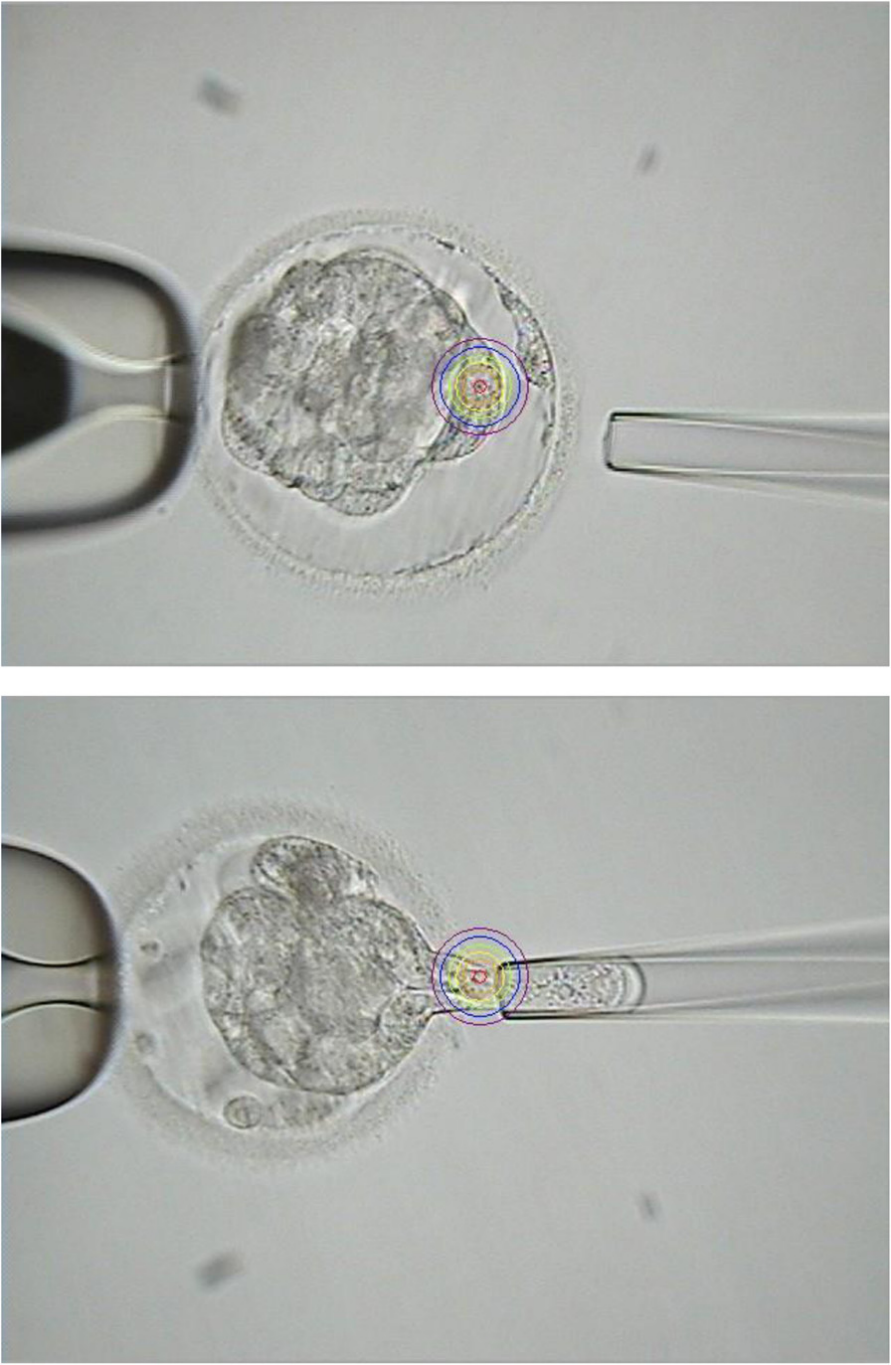
Images of blastocyst biopsy after cryopreservation and warming. Top image indicates the blastocyst before biopsy and bottom image indicates blastocysts during biopsy

If the blastocysts survived after warming, they were biopsied as per the following procedures (also showed in Fig. 1):

Blastocysts were transferred from a culture dish to a biopsy dish in which 20–30 μl drops of biopsy medium were previously made and covered with tissue culture oil. The dish was then moved to a warmed inverted phase contrast microscope stage and the biopsy was performed at × 400 magnification. For biopsy, the blastocyst was held from left side (9 o’clock position) and a ~20 μm hole was made in the zona pellucida on the right side (3 o’clock position) using the ZILOS-tk™ laser system at a medium pulse power. Typically, 2–3 pulses were required to make the zona pellucida open completely. Because blastocysts have a large perivitelline space (Fig. 1) after warming, zona opening by laser rarely damages the embryo proper.

After opening the zona pellucida, a biopsy pipette (20 μm diameter) was inserted into the opening in the zona and cells were aspirated from the blastocysts. After a few cells were aspirated into the biopsy pipette, the biopsy pipette was pulled outside of the zona and at the same time, a laser (low power or medium power) was used to cut the cell connection in the front of the tip of biopsy pipette. Three to five pulses were necessary to separate some cells from the embryo proper. Approximately 5 ~ 10 TE cells were biopsied from the embryos.

After biopsy of a maximum of 4 blastocysts each time, the biopsy dish was moved to a workstation and TE cells from each sample were individually transferred into properly labeled microcentrifuge tubes containing microarray buffer under a stereomicroscope. The embryo proper was transferred to culture dishes after washing with culture medium 3–5 times, and then the culture dish was returned to the incubator for continuing culture until the day (second day morning) for transfer. The biopsied samples were then subjected to whole genome amplification (WGA) for 24-chromosomal analysis by microarray [19, 20].

### Sample amplification

For DNA extraction and fragmentation, TE cell samples and negative controls were collected in 2 μl of PBS buffer, lysed with 2 μl of SurePlex cell extraction buffer and 5 μl of the SurePlex Extraction cocktail master mix and then incubated at 75 °C for 10 min followed by further incubation at 95 °C for 4 min. Genomic DNA (gDNA) was randomly fragmented by adding 5 μl of SurePlex Pre-amplification cocktail to the lysed biopsy samples or to gDNA controls and the mixture was incubated according to the following protocol: one cycle of 95 °C for 2 min, followed by 12 cycles of 95 °C for 15 s, 15 °C for 50 s, 25 °C for 40 s, 35 °C for 30 s, 65 °C for 40 s and 75 °C for 40 s, followed by a hold at 4 °C.

Genomic DNA was amplified using the PicoPLEX WGA Kit (NEB) according to the following thermal cycler program: one cycle of 95 °C for 2 min, followed by 14 cycles of 95 °C for 15 s, 65 °C for 1 min and 75 °C for 1 min, followed by a hold at 4 °C. Successful amplification was confirmed by electrophoresis of 5 μl of each amplified sample plus 5 μl gel loading buffer on a 1.5 % agarose TBE gel.

### aCGH of gDNA

For aCGH, 13 μl of amplified samples were labeled with Cy3 or Cy5 using SureTag DNA labeling kit and labeled samples were combined and co-precipitated with COT Human DNA in preparation for hybridization. Labelled DNA was then resuspended in dextran sulphate hybridization buffer and loaded onto SurePrint G3 human CGH 8 × 60 K oligo microarrays following manufacturer’s protocol. Reference DNA (both male and female were used) for array was obtained from Promega (Promega Corporation, Madison, WI USA).

Labelled products were then hybridized to array slides. After hybridization, array slides were washed to remove unbound labelled DNA and scanned with SureScan scanner at 3 μM to excite the hybridized fluorophores read and store the resulting images of the hybridization. Finally, scanned images were analyzed by Cytogenomics 2.7.8.0 software following manufacturer’s protocol. Human Genome Build 19 (hg19) was used in the present study.

### Patient preparation for embryo transfer

All patients for embryo transfer received estradiol orally and transvaginally. Intramuscular administration of progesterone oil was initiated after about 14 days of estradiol treatment. Endometrium thickness was measured on the day of progesterone administration. After PGS, if the patients had normal euploid blastocysts, embryo transfer was performed on the sixth or seventh day of progesterone administration and progesterone was continued until the first serum β-hCG test two weeks after transfer. Aneuploid embryos were discarded after acquiring consents from patients or re-vitrified with other remaining euploid blastocysts. Ongoing pregnancies were supported by continued estradiol and progesterone.

### Pregnancy and live birth/ongoing rate assessment

Fourteen days after embryo transfer, pregnancy was tested by a serum β-hCG assay. When the β-hCG was > 5 mIU/mL the patients were regarded as having a biochemical pregnancy. Four weeks after embryo transfer, when a gestational sac and a heartbeat appeared ultrasonographically, the patients were diagnosed as having a clinical pregnancy. The live birth rate and ongoing rate were indicated as live birth or ongoing per embryo transfers.

## Results

As shown in Table 1, 234 blastocysts were warmed from 35 warming cycles including 17 patients at ages of ≤35 years old (young patients), 16 patients at ages of ≥36 years old (advanced maternal age) and 2 recipients using donated eggs. All of these patients had previous embryo implantation failures (1–4 times), so they decided to screen their embryos before FET. Due to the limited number of patients in these groups, statistical analysis between groups was not performed, and the major objective was focused on the method assessment.

**Table 1:** Clinical summary of application of PGS on frozen/warmed blastocysts

Age (range)	≤35 (28–34)	≤36 (36–44)	Egg donor	Total
No. of cases	17	16	2	35
No. of embryos warmed	121	98	15	234
No. (%) of embryos survived	119 (98.3)	60 (91.8)	15 (100)	224 (95.7)
No. of embryos biopsied	119	90	15	224
No. (%) of samples without test results	1 (0.8)	1 (1.1)	1 (6.7)	3 (1.3)
No. (%) of embryos with test results	118 (99.2)	89 (98.8)	14 (93.3)	221 (98.7)
No. (%) of normal embryos	72 (61.0)	51 (57.3)	8 (57.1)	131 (59.3)
No. (%) of abnormal embryos	46 (38.9)	38 (42.7)	6 (42.9)	90 (40.7)
No. of transfer	17	15^a^	2	34
No. (%) of clinical pregnancy	9 (52.9)	7 (46.7)	2 (100)	18 (52.9)
No. of delivery and ongoing pregnancy	6 (35.3)	6 (40.0)	2 (100)	14 (41.2)
No. of embryos transferred	32	25	4	61
No. (%) of embryos implanted	13 (40.6)	10 (40.0)	3 (75.0)	26 (42.6)

^a^One patient did not have transfer due to all abnormal embryos

After warming, 224 (95.7 %) blastocysts survived and then were biopsied. After aCGH, 3 (1.3 %) blastocysts did not have test results due to fragmented DNA or low amount of DNA, and 221 (98.7 %) samples had diagnostic results. Out of these samples, 59.3 % were normal euploid and 40.7 % had chromosomal anomalies, including aneuploidies, partial chromosome deletion and/or duplication (Table 2). As shown in Table 2, most samples (65.6 %) had single chromosomal abnormalities, while 34.4 % samples had multiple chromosomal errors, i.e. at least two chromosomes had errors.

**Table 2:** Chromosome distribution in abnormal embryos

Chromosomes	No. of samples analyzed	No. of aneuploid samples	No. of euploid with deletion	No. of euploid with duplication	No. of euploid with deletion & duplication
Single abnormality	59 (65.6 %)	42 (71.2 %)	13 (22.0 %)	2 (3.4 %)	2 (3.4 %)
Multiple abnormalities	31 (34.4 %)	29 (93.6 %)	0	0	2 (6.5 %)
Total	90	71 (78.9 %)	13 (14.4 %)	2 (2.2 %)	4 (4.4 %)

**Fig. 2:**
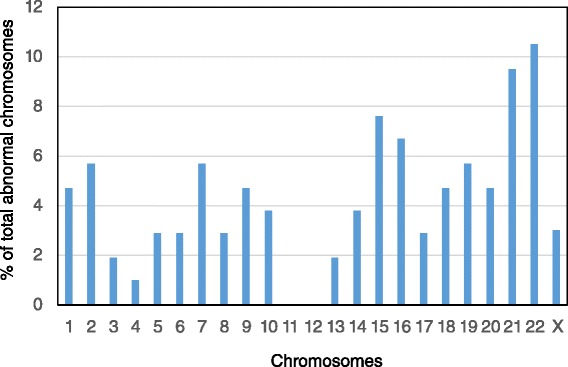
Distribution of abnormal chromosomes in the aneuploid blastocysts detected by TE biopsy and DNA microarray

As shown in Fig. 2, chromosome abnormalities were observed in most chromosomes except chromosomes 11 and 12. Errors in chromosomes 21 and 22 were the most frequent chromosome anomalies, followed by chromosomes 15, 16, 2, 7 and 19.

During the study period, 34 out of 35 patients had at least one euploid blastocyst for transfer, and 52.9 %, 46.7 % and 100 % (2/2) of clinical pregnancy rates were obtained in patients at ages of ≤ 35, ≥36 and those who received donor eggs, respectively, with an overall 52.9 % of pregnancy rate and 42.6 % of embryo implantation rate. Eight patients delivered normal babies and six still had ongoing pregnancy.

## Discussion

The present study, for the first time, reported the efficiency of frozen/warmed blastocyst biopsy for PGS. Our data showed that frozen/warmed blastocyst biopsy can be done within an hour after completion of warming, and FET can be done within 24 h after warming if there are normal embryos. Because biopsy and aCGH take more than 12 h, FET was scheduled on the second day after embryo warming and biopsy. If the analysis could be done in a shorter time, FET could be scheduled on the same day for the patients. However, based on our experience, it would appear that embryo quality was not affected by extended culture up to 24 h. It has been reported that transfer of frozen/warmed blastocysts after overnight culture (less than 18 h) had similar implantation rates as compared with transfer of blastocysts after same day warming [16].

Usually blastocysts start to expand 2–3 h after completion of warming, and it may be easier to choose the location for biopsy if blastocysts re-expand. However, this would take more time from embryo warming to FET and it may be unfavorable to blastocyst implantation. From the results obtained in the present report, we found that our method is practical. It is still not known if biopsy of re-expanded blastocysts can further improve clinical outcomes.

For the warmed blastocyst biopsy, it is not necessary to warm all blastocysts if patients have many cryopreserved embryos in order to avoid re-freezing spare blastocysts after transfer. Although reports indicated that refreezing human blastocysts did not further impair embryo quality and implantation [19–22], repeated cryopreservation and warming may be not a good practice for human IVF, as long term effects of refreezing/warming on human embryos are not clear. Only limited case reports indicated that embryo survival was not affected by these procedures [19]. Therefore, in order to avoid repeated freezing/warming, it may be necessary to warm a limited number of embryos for biopsy and analysis. The only concern of testing a limited number of blastocysts is that there may not be normal blastocysts for transfer after analysis, thus embryo transfer has to be cancelled. In the present study, some patients had all frozen blastocysts warmed and analyzed while some patients had a portion of blastocysts warmed and analyzed. As a result, only one patient did not have a euploid embryo, so her FET was cancelled, while all others had at least one normal embryo for FET.

Another concern is that there are limited number of good quality of normal blastocysts for transfer if a portion of embryos are warmed and analyzed. Because some euploid blastocysts may have fair or poor quality. It has been found that embryo implantation is related to the total available embryos for PGS, which would ensure that there is at least one good blastocyst to be euploid for transfer [23]. Morphology and euploid status are the two most important factors affecting embryo implantation. Several studies have shown that morphology assessment alone to select embryos for transfer has limited prediction capacity for embryo implantation, as some high morphology score embryos may be aneuploid [7, 24, 25], especially in patients of advanced maternal age. Also, aneuploid embryos with good morphology may result in miscarriage. Therefore, high live birth rate should be obtained after transfer of embryos that have high morphology score and are euploid.

In the present study, all embryos were biopsied at the blastocyst stage. Many studies have proven that blastocyst stage is the optimal stage to perform biopsies for PGS, which produces higher implantation rates and provides more reliable aneuploidy results than cleavage stage embryos [13, 14, 26]. Biopsy at blastocyst stage does not result in a loss of inner cell mass and would not affect subsequent fetal development, so it is less harmful to embryo development [14, 15]. Furthermore, blastocyst biopsy can acquire multiple TE cells [27], thus the results are more accurate.

It has been repeatedly reported that more than half of all morphologically normal blastocysts in women >35 years old are aneuploid [7, 8, 12], and more than 50 % of first-trimester spontaneous abortions are chromosomal aneuploid [28]. So by doing PGS, euploid embryos can be selected for transfer, thus implantation rates can be improved, and miscarriages and trisomy offspring loss can be reduced [11, 12].

As for the prevalence of aneuploidy and degree of chromosome anomalies in the cryopreserved/warmed blastocysts, it would appear that there was not much difference between fresh blastocysts and frozen blastocysts. In the present study, we found that the overall aneuploidy rate was 40.7 %, which was similar to that in our previous report with fresh blastocyst aCGH [8]. However, direct comparison is difficult because patients’ populations are different between studies. Another similar result was the degree of chromosome anomalies in the aneuploid embryos. In the previous study with fresh blastocysts, it was found that 62.3 % of aneuploid blastocysts had single chromosome errors [8], while the rate was 65.6 % in the present study with frozen/warmed blastocysts. Also, it was found that chromosomes 21 and 22 were two of the most frequent chromosome abnormalities in the aneuploid blastocysts in the fresh blastocyst [8] as well as in the frozen/warmed blastocysts (current study). Again, although the comparison is difficult between studies, the results may suggest that the prevalence of aneuploidy in fresh blastocysts and frozen blastocysts would be similar and cryopreservation/warming may not increase chromosome abnormalities in human blastocysts.

In the present study, our goal was limited to examine the feasibility of biopsy of cryopreserved blastocyst for PGS. We did not compare the outcome between PGS and non-PGS cases, so the necessity of PGS on all previous IVF failures is still debatable as other factors (not only embryo ploidy) also affect embryo implantation, such as endometrium preparation and transfer itself. All IVF failures cannot be attributed to embryonic aneuploidy. As indicated in two recent review papers [29, 30], chromosomal mosaicism may cause incorrect embryo screening, especially diploid-aneuploid mosaicism, which is the most common chromosome mosaic in human embryos [29], thus some screened “abnormal” embryos may have normal inner cell mass [8, 31]. Transfer of these kinds of embryos may produce normal healthy babies. Indeed, recently, Greco et al. reported that transfer of mosaic aneuploid blastocysts can result in a high healthy live birth rate (33 %) [32]. In this report, some of the embryos transferred had two chromosomal abnormalities [32]. One of our patients also had a healthy baby after transfer of a screened embryo, but this pregnancy had a mosaic placenta although PGS showed it was a euploid [33]. These results indicate that mosaic embryos can produce healthy babies. As suggested by Liu et al., if all embryos are aneuploidy in an IVF cycle, a second biopsy may be necessary to find a transferrable embryo [8]. However, according to Greco et al.’s report [32], transfer of these “aneuploid’ embryos after additional consents were signed by the patients may be practical without a second biopsy that may cause more damages to embryos. Therefore, a careful discussion and decision with patients should be necessary if the patients do not have a normal embryo for transfer after PGS.

It has been reported that PGS can also improve clinical outcome in young patients, especially when eSET is applied [10]. In our previous study, we did not find significant increase in either clinical pregnancy or embryo implantation when blastocysts from donated eggs were screened and transferred, especially when two blastocysts were transferred. As suggested by these authors, unrestricted clinical application of current PGS (either aCGH or NGS) should be avoided [30, 31, 34].

In the present study, we found that 65.6 % of aneuploid samples had single chromosomal error. If diploid-aneuploid mosaic rate is high in these embryos as indicated in the previous study (59 %) [29], it would be possible that transfer of these embryos can produce normal pregnancy and result in healthy live births as reported by Greco et al. [32]. Thus it must be careful to make the decision to transfer, cryopreserve or discard these embryos. As hypothesized by Greco et al. [32], the extent and type of mosaicism may affect the IVF success rate, and additional studies with large sample size are necessary to test this hypothesis.

## Conclusions

From the present study, we conclude that frozen blastocysts can be warmed and biopsied for aneuploidy screening. The normal euploid blastocysts can be transferred within 24 h after warming, and embryo implantation should not be impaired by the procedures. Also, the prevalence of aneuploidy in human blastocysts does not increase after blastocysts are cryopreserved and warmed, suggesting that current blastocysts vitrification and warming methods are safe. As thousands of human embryos have been cryopreserved without PGS, it may be necessary to perform biopsy and PGS if the patients had repeated previous implantation failures, miscarriages, and/or are of advanced maternal age.

## Abbreviations

aCGH: array comparative genomic hybridization
FET: frozen embryo transfer
hCG: human chorionic gonadotropin
IVF: in vitro fertilization
NGS: next generation sequencing
PGS: preimplantation genetic screening
TE: trophectoderm
WGA: whole genome amplification
